# Role of multidetector computed tomography in evaluating complications following endovascular repair of aortic aneurysm

**DOI:** 10.4103/0975-3583.70907

**Published:** 2010

**Authors:** Zarina Abdul Aziz, Purushotham R. Naidu, Jagadish Prasad, Arjun Kalyanpur

**Affiliations:** *Narayana Institute of Health Sciences and Teleradiology Solutions, Bangalore, India*

**Keywords:** Aneurysm, endoleaks, multidetector computed tomography, stents

## Abstract

**Objective:**

To study the role of multidetector computed tomography (MDCT) in evaluating various complications following endovascular stenting of aortic aneurysms.

**Materials and Methods:**

Over a period of 2 years (June 2005 to June 2007), 50 patients with aortic aneurysm on computed tomography (CT) angiogram were prospectively studied. Images were acquired on a 64 slice multidetector row CT scanner (GE—LightSpeed VCT) after intravenous administration of nonionic iodinated contrast. Nineteen patients underwent endovascular stent-graft repair based on their medical and surgical risk factors. Stent-graft related complications were recorded by CT angiography and analyzed using descriptive statistics.

**Results:**

Most common complication related to the endovascular stent-graft placement was endoleak (44.4%), followed by puncture site hematoma (27.8%), thrombotic occlusion of a limb of the bifurcated stent graft, kinking of the stent-graft, and difficult catheterization with intimal tear in the common iliac artery were 5.6% each. Poststent diameter of the aneurysm was an important predictor of endoleaks. All the patients with either increase or no change in the aneurysm size had endoleaks.

**Conclusion:**

MDCT angiography is an important modality in identifying, describing, and following up the various complications following endovascular repair of aortic aneurysms, endoleaks being the most common complication. Decrease in the poststent diameter of the aneurysm suggested a good outcome.

## INTRODUCTION

Open surgical repair is considered the definitive treatment for diseases of the thoracic and abdominal aorta. It necessitates significant recovery time and also carries the risk of increased morbidity and mortality in the elderly patients with multiple concurrent diseases. A study by Therasse *et al*. stated a mortality rate of 7–12% in elective cases and up to 40% in emergent situations.[1] It has also been mentioned by many authors that by eliminating the more invasive surgical procedures, the mortality and morbidity could be potentially decreased with improved recovery time.[2–4] With the development of minimally invasive surgical techniques, endovascular stent-graft placement has become an accepted and widely used alternative to the traditional surgical repair of aortic disease and is gaining acceptance as treatment of choice.

Common aortic pathologic conditions amenable to stent placement include aortic aneurysm, pseudoaneurysm, penetrating ulcer, congenital abnormalities, and aortic dissection. Over the past decade, the endovascular repair of aortic aneurysm has widely replaced the conventional surgery. This technique has been reported to have a lower mortality rate, a shorter hospital stay, and less surgical morbidity. Endovascular repair is not free of complications. Endoleak being the common and unique complication of endovascular repair and its persistence represents a failure of the endovascular treatment. Accurate detection and classification of endoleaks is essential for its proper management.[5]

Unlike open surgical repair of aortic aneurysm, successful completion of endovascular repair largely depends on medical imaging. Computed tomography angiography (CTA) has been confirmed by many studies to be the preferred modality in both preoperative planning and postoperative follow-up of endovascular aortic repair.

MDCT has been reported to be superior to single slice CT in nearly all clinical applications including surface shaded display (SSD), maximum-intensity projection (MIP), curvilinear reformation (CVR), volume rendering (VR) and virtual intravascular endoscopy (VIE). Many studies have been performed to assess the value of MDCT following endovascular repair.

In this article, we demonstrate how MDCT with twodimensional (2D), multiplanar reformation (MPR), and three-dimensional (3D) rendering is relevant in postoperative assessment of endovascular aortic aneurysm repair. We describe the spectrum and frequency of various complications following endovascular repair of aortic aneurysm and discuss whether CT angiogram can predict the success of such endovascular stent repair.

## MATERIALS AND METHODS

In total, 50 consecutive patients with aortic aneurysm of either sex aged between 15 and 90 years referred to the Department of Radiology for CTA of the aorta were included in this study over the period of June 2005 to June 2007.

Out of the total, 19 patients were subjected to endovascular stent repair based on the favorability for endovascular accessibility, location, and size of the aneurysm as well as absence of associated complications such as rupture or impending rupture. The prestenting CT (in all the 50 patients) was performed approximately 2 weeks to 1 month prior to the endovascular repair.

Poststenting (in 19 patients including the follow-up) images were performed during acute (0–7 days), intermediate (1 week to 6 months), and late (>6 months) periods. Images were acquired on a multidetector 64 slice CT scanner (GE—LightSpeed VCT) after intravenous administration of nonionic iohexol 350 (1.5–2 mL/kg) injected at the rate of 3.5 mL/s by power injector using Smartprep technique, with the region of interest (ROI) on the descending thoracic aorta.

Scan parameters used were as follows: Detector coverage: 40 mm; slice thickness: 5 mm, reconstructed into 0.625 mm; Pitch and speed (mm/rot): 1.375:1, 55.0; rotation time: 0.5 sec.

**Figure 1:**
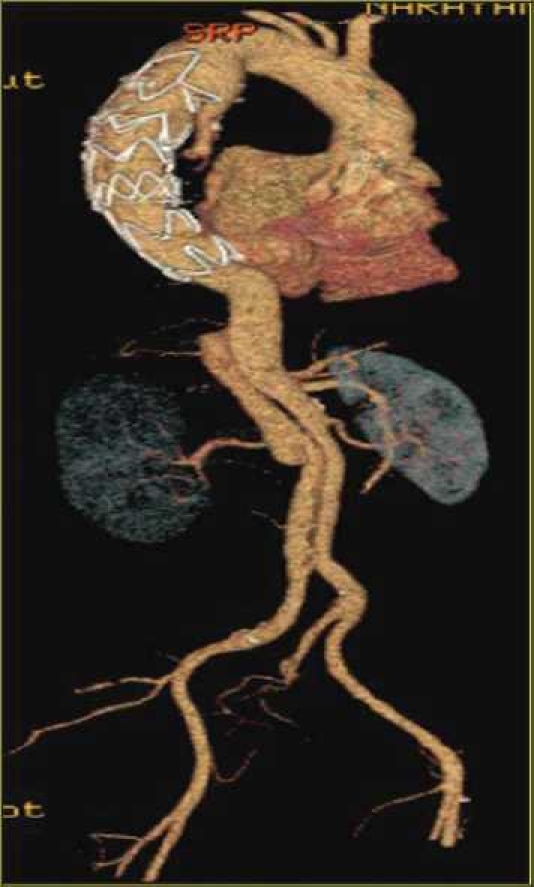
Volume rendered 3D image showing the placement of an endovascular graft in the repair of a descending thoracic aortic aneurysm, its extent and diameter.

KV: 120; MA: 500–650; algorithm: standard; Window setting: mediastinum; area covered: root of neck to inguinal regions; scan direction—cranio caudal; Recon 1 Filter: standard; Recon 1 Type: SnapShot Segment; 3D reformats; MIP, and VR were performed in all examinations [Figure 1].

Stent-grafts used: 12 patients had medtronic stents and 7 had talent grafts. Data were analysed using SPSS 15.0, Stata 8.0, Medical 9.0.1, and Systat 11.0 and results are represented in the form of graphs and tables.

## RESULTS AND ANALYSIS

Fifty patients were enrolled in this study. The mean age of the patients was 54.50 years (range, 36.71–72.79 years). Most common age group was fifth decade accounting for 26% of patients enrolled. There was a clear male preponderance with 80% of the study cohort belonging to this sex. Out of the total, 19 patients were subjected to endovascular repair and two underwent surgical repair (one of those patient died during the surgical repair). Twentyeight patients were subjected to regular interval follow-up CT angiograms, quarterly, half-yearly, or annually as decided by the treating surgeon/physician. One patient died due to aneurysm-related acute complications (i.e., aneurysm rupture with large retroperitoneal hematoma).

Of the 19 patients who underwent endovascular repair, seven were diagnosed with aneurysm of thoracic aorta, 11 with abdominal aortic aneurysm and one with thoracoabdominal aortic aneurysm.

In total, 28 poststenting follow-up CT angiograms were taken in 19 patients, four (14.2%) patients were imaged in the acute period (0–7 days), 18 (64.2%) in the intermediate period (1 week to 6 months) and 6 (21.4%) in the late period (>6 months) [Table 1]. The poststent dimensions of the aneurysm, the dimensions of the stent used, location of the stent, and distance of the proximal end of the stent from the nearest major branch artery were described.

**Table 1:** Correlation between the pre- and poststent aneurysm diameter (outer wall to outer wall)

Site of stent graft	Prestent CTA (cm)	Poststent CTA (cm)		
		Acute (1–7 days)	Intermediate (1 week–6 months)	Late (>6 months)
Infrarenal AAA	4.2 × 3.6	4.1 × 3.5	4.3 × 3.7	
Infrarenal AAA	7.3 × 5.9		6.7 × 4.3	
Infrarenal AAA	8.0 × 5.3		7.8 × 5.0	
Infrarenal AAA	6.7 × 5.8		6.9 × 5.8	6.5 × 4.9
Infrarenal AAA	4.5 × 3.7		4.1 × 3.5	
Infrarenal AAA	3.8 × 2.2	3.9 × 3.0	3.6 × 2.3	
Infrarenal AAA	7.0 × 6.1		7.0 × 5.9	6.6 × 5.5
TA	5.7		5.6	5.4
TA	5.3 × 4.3		5.4 × 4.3	
TA	4.8 × 3.2		5.0 × 3.3	
TA	5.6 × 5.9		5.6 × 6.0	5.5 × 6.1
TA	7.3 × 6.5		7.0 × 6.3	
TA	6.7		6.7	
TA	5.6 × 4.3	5.5 × 4.2		5.3 × 4.0
TA	5.7		5.6	5.4
TA	8.0 × 5.3		7.7 × 5.0	
TA	3.8 × 2.2	3.9 × 3.0	3.7 × 2.1	
TA	7.3 × 6.5		7.0 × 6.3	
TAA	5.7 × 4.2	−	5.8 × 4.3	−
Increased from prestent CTA			5	−
No change from prestent CTA			1	−
Decreased from prestent CTA			12	6
Total* (28)			18 (64.2%)	6 (21.4%)

* Total 28-poststenting follow-up CT angiograms were taken in 19 patients. AAA = abdominal aortic aneurysm; TA = thoracic aortic aneurysm; TAA = thoracoabdominal aortic aneurysm.

Overall, there was no mortality related to the endovascular procedure. There was no major acute endovascular repair procedure-related morbidity in all the cases studied.

**Figure 2:**
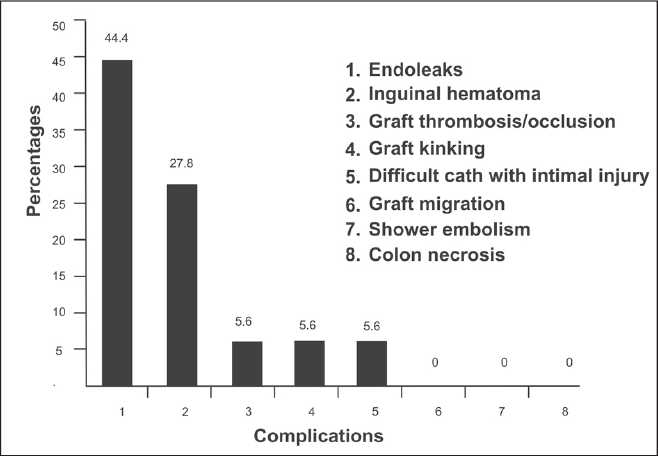
Complications related to endovascular stenting.

**Figure 3:**
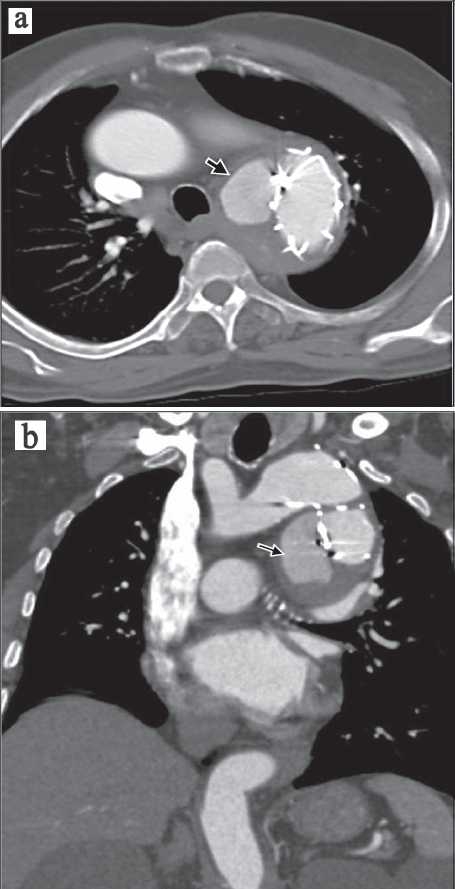
(a and b) Type I endoleak from the proximal site of graft anastomosis in a 46-year-old male after 1 month of endovascular repair of the descending thoracic aneurysm.

**Figure 4:**
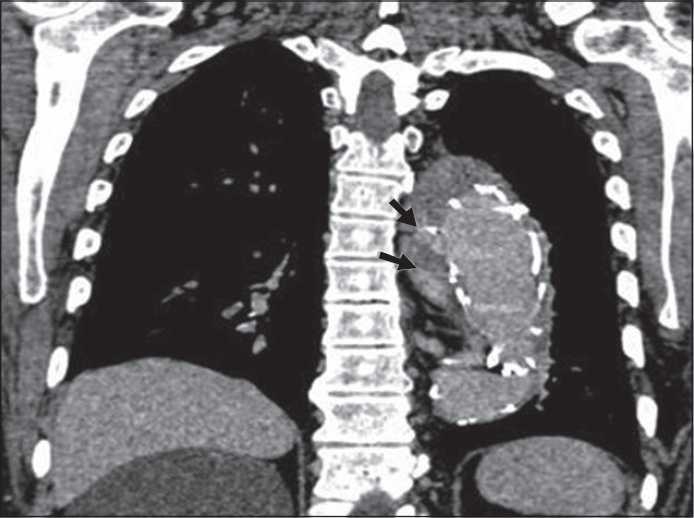
Type III endoleak, i.e., blood flow into the aneurysm sac due to inadequate or ineffective sealing of overlapping graft joints or rupture of the graft fabric. rupture of the graft fabric.

**Figure 5:**
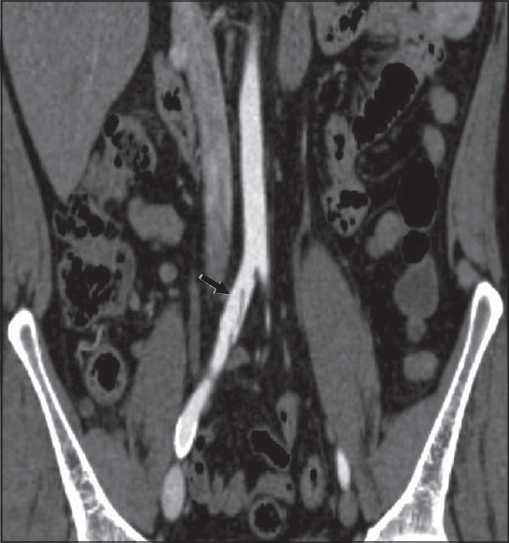
Difficult catheterization during the procedure with resultant intimal tear in the right common iliac resulting into acute dissection.

The most common complication related to the endovascular stent placement [Figure 2] was endoleak accounting for 44.4% (8 patients, 10 endoleaks). The endoleaks were classified as proposed by White *et al*.[6] Out of the 10 endoleaks, seven were of type I [Figure 3a and b], two were of type II, and one was of type III [Figure 4]. No cases of types IV and V endoleaks were seen in the study. One case of thrombotic occlusion of the right limb of the bifurcated stent-grafts was identified. Another case demonstrated stent kinking during the intermediate period. One patient had a difficult catheterization during the procedure with a resultant intimal tear in the right common iliac artery resulting in acute dissection [Figure 5]. This was managed by placing a separate stent in the dissected vessel. None of the patients who underwent stent-graft placement demonstrated other proceduresrelated complications such as paraplegia, stroke, or major visceral infarction. Another common, but less life-threatening complication seen with this procedure was puncture site hematoma, accounting for 27.8% cases. These were managed conservatively [Figure 4].

**Figure 6:**
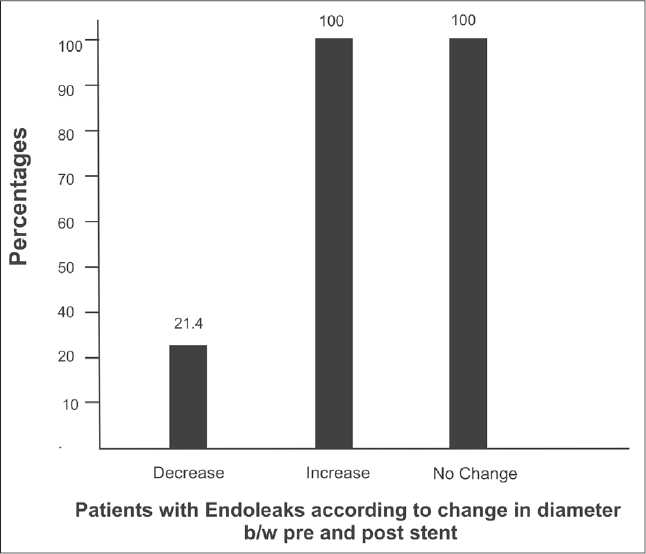
The correlation of change in diameter of the aneurysm with the presence of endoleaks.

Poststent interval increase in transverse dimension of the aortic aneurysm (i.e., outer wall to outer wall) supports the presence of endoleaks; and is felt to be endovascular stenting [Figure 6]. We analysed the change in the poststenting diameter of the aneurysm and checked whether it was associated with the presence or absence of endoleaks. All the patients with either increase or no change in the aneurysm size had endoleaks. On the contrary, only 21.4% of the patients showing interval reduction in the size demonstrated the presence of endoleaks. Other observations were that the only patient with a thoracoabdominal stent-graft showed the presence of an endoleak, 5 out of 11 with thoracic stent-grafts showed endoleaks, and 2 out of 7 with infrarenal abdominal stentgrafts showed endoleaks [Table 2].

**Table 2:** Association of site of stent-graft and endoleaks

Site of stent graft/ type of aneurysm	Number of patients	Endoleaks	
		Absent	Present
Site of stent-graft Infrarenal AAA	7	5	2
TA	11	6	5
TAA	1	0	1

Above mentioned results support that CT angiogram can predict the success of endovascular stent repair.

## DISCUSSION

Endovascular management is becoming an important modality of treatment of aortic aneurysms and is likely to replace conventional open surgical repair in the near future due to its minimally invasive nature. Endovascular repair of aortic aneurysms aims to exclude the aneurysmal sac from circulation without using laparotomy, by accurately placing a stent-graft by means of the transfemoral approach. It requires accurate modeling of the aorta and aneurysm to select a stent-graft with appropriate dimensions for each individual patient and to plan insertion prior to the procedure. MDCT has been confirmed to be the preferred modality in both preoperative planning and postoperative follow-up of endovascular aortic repair. It is also considered the standard of reference in the detection of endoleaks.[6]

The various complications described in relation to endovascular treatment of aortic aneurysms include endoleaks[6] (type I to type V endoleaks), graft thrombosis,[7,8] graft kinking,[9–11] pseudoaneurysm caused by graft infection,[8,12,13] graft occlusion,[8,14,15] shower embolism,[16,17] colon necrosis,[11,18] aortic dissection,[19] and hematoma at the arteriotomy site.[20] Out of the many advantages of MDCT, the few worth mentioning in this context are the better visualization of true and false luminal flow channels, intramural hematomas communicating with the aortic lumen, and slow perigraft flow around aortic stentgrafts. In our study, we analyzed the various complications following endovascular stenting of aortic aneurysms using MDCT. This study demonstrated that endoleaks are the most common postendovascular repair complications accounting for 44.4% of the cases, with type I endoleaks being the most common. Furthermore, poststent interval decrease in transverse dimension of the aortic aneurysm correlated well with the absence of endoleaks; hence, this remains an important parameter in depicting the outcome of endovascular stenting. The results of this study is in accordance with the study conducted by Marcheix *et al*.,[21] which detected 42% primary endoleaks. Dake *et al*.[22] also found that, in the absence of a detectable endoleak, the size of thoracic aneurysms decreased in 48% of patients, were unchanged in 22%, and increased in 22% at a mean follow-up of 1.1 years.

The key limitation of our study is that the MDCT findings were not directly correlated with the catheter angiogram findings. Also, not all the postrepair patients were imaged and followed up at regular intervals. Postoperative lifelong follow-up with CT and high-quality imaging continue to be essential for all patients after endovascular repair. Additional studies on a larger scale are, therefore, required to further substantiate these results.
